# Impacts of Atmospheric CO_2_ and Soil Nutritional Value on Plant Responses to Rhizosphere Colonization by Soil Bacteria

**DOI:** 10.3389/fpls.2018.01493

**Published:** 2018-10-22

**Authors:** Alex Williams, Pierre Pétriacq, David J. Beerling, T. E. Anne Cotton, Jurriaan Ton

**Affiliations:** ^1^Department of Animal and Plant Sciences, University of Sheffield, Sheffield, United Kingdom; ^2^P^3^ Institute for Translational Plant and Soil Biology, Department of Animal and Plant Sciences, University of Sheffield, Sheffield, United Kingdom; ^3^UMR 1332 Fruit Biology and Pathology, INRA-Bordeaux & University of Bordeaux, Villenave d’Ornon, France; ^4^Plateforme Métabolome du Centre de Génomique Fonctionnelle de Bordeaux, INRA – Bordeaux, Villenave d’Ornon, France

**Keywords:** CO_2_, PGPR, global change, rhizosphere, ISR

## Abstract

Concerns over rising atmospheric CO_2_ concentrations have led to growing interest in the effects of global change on plant-microbe interactions. As a primary substrate of plant metabolism, atmospheric CO_2_ influences below-ground carbon allocation and root exudation chemistry, potentially affecting rhizosphere interactions with beneficial soil microbes. In this study, we have examined the effects of different atmospheric CO_2_ concentrations on Arabidopsis rhizosphere colonization by the rhizobacterial strain *Pseudomonas simiae* WCS417 and the saprophytic strain *Pseudomonas putida* KT2440. Rhizosphere colonization by saprophytic KT2440 was not influenced by sub-ambient (200 ppm) and elevated (1,200 ppm) concentrations of CO_2_, irrespective of the carbon (C) and nitrogen (N) content of the soil. Conversely, rhizosphere colonization by WCS417 in soil with relatively low C and N content increased from sub-ambient to elevated CO_2_. Examination of plant responses to WCS417 revealed that plant growth and systemic resistance varied according to atmospheric CO_2_ concentration and soil-type, ranging from growth promotion with induced susceptibility at sub-ambient CO_2_, to growth repression with induced resistance at elevated CO_2_. Collectively, our results demonstrate that the interaction between atmospheric CO_2_ and soil nutritional status has a profound impact on plant responses to rhizobacteria. We conclude that predictions about plant performance under past and future climate scenarios depend on interactive plant responses to soil nutritional status and rhizobacteria.

## Introduction

Atmospheric CO_2_ influences microbial biomass and diversity in the rhizosphere (Paterson et al., 1997). The plant-mediated effects of atmospheric CO_2_ on soil microbial communities are well documented (Wiemken et al., 2001; Montealegre et al., 2002), indicating a dominant, plant-mediated mechanism. It is likely that the variation in root- microbe interactions under different atmospheric conditions are due to changes in root exudates which are estimated to contain between 5 and 40% of plant photosynthetically fixed carbon (Lynch and Whipps, 1990; Hinsinger et al., 2006; Marschner, 2012). Since rhizodeposition of carbon (C) increases under elevated CO_2_ (*e*CO_2_; Phillips et al., 2009; Eisenhauer et al., 2012), it can be expected that rhizosphere colonization by microbes relying on C from plant exudates will also be enhanced (Lipson et al., 2005; Kassem et al., 2008; Eisenhauer et al., 2012). While it is clear is that CO_2_ alters overall microbial community composition across a range of different soil-types (Montealegre et al., 2002; Janus et al., 2005), the extent to which *e*CO_2_ affects microbial interactions in the rhizosphere remains controversial. Using chloroform fumigation extraction to estimate microbial biomass, previous studies have reported both positive and negative relationships with *e*CO_2_ (Rice et al., 1994; Ross et al., 1995; Kassem et al., 2008; Eisenhauer et al., 2012). It also remains contentious in how far *e*CO_2_ induces shifts between fungal or bacterial communities, and the resultant effects on the functioning on rhizosphere microbes (Ross et al., 1995; Lipson et al., 2005; Drigo et al., 2008).

Early research on plant growth responses and the presence of specific rhizosphere microbes to *e*CO_2_ have suggested a possible relationship between *e*CO_2_, plant growth and increases in colonization by plant growth-promoting rhizobacteria (PGPR; O’Neill et al., 1987). PGPRs are often closely associated with plant roots and should, therefore, be more reliant on plant-derived C (Denef et al., 2007). Although many studies have addressed the effects of *e*CO_2_ on plant-rhizobia and plant-mycorrhiza interactions (e.g., Rogers et al., 2009; Mohan et al., 2014), little is known about the specific impacts of *e*CO_2_ on PGPR (Drigo et al., 2008). Considering that PGPR modulate a range of agronomically important plant traits, including plant growth, abiotic stress tolerance and resistance to pests, and diseases (Lugtenberg and Kamilova, 2009), this knowledge gap limits our ability to predict how anthropogenic global change will impact crop production and food security. Furthermore, the impacts of CO_2_ across a range of CO_2_ conditions, including sub-ambient CO_2_ (*sa*CO_2_), remain poorly documented (Field et al., 2012). In a CO_2_ gradient study (200–600 ppm), microbial biomass and soil respiration from a grassland ecosystem were not clearly related to CO_2_ concentration (Gill et al., 2006). By contrast, analysis of fungal communities, using pyrosequencing of internal transcribed spacer sequences, revealed a positive relationship between operational taxonomic unit richness and CO_2_ concentration that was soil-type dependent (Procter et al., 2014). While these studies suggest that atmospheric CO_2_ impacts on plant-beneficial microbes in the rhizosphere, it remains difficult to ascertain the underpinning mechanisms and predict the corresponding plant responses to altered colonization by these microbes. Most studies on the effects of CO_2_ gradients on rhizosphere microbes involved field experiments, which are prone to environmental variability, such as nutrient availability, soil moisture, temperature, soil pH, and plant species present (Freeman et al., 2004; Castro et al., 2010; Classen et al., 2015; Dam et al., 2017) and do not allow the manipulation of bacteria in the rhizosphere, hence preventing examination of their function.

In this study, we have investigated the impacts of a pre-industrial concentration of *sa*CO_2_ and a worst-case scenario projected concentration of *e*CO_2_ on rhizosphere colonization of Arabidopsis roots by two well- characterized soil bacteria: the rhizosphere colonizer *Pseudomonas simiae* WCS417 (previously named *Pseudomonas fluorescens* WCS417; Berendsen et al., 2015) and the saprophytic soil colonizer *Pseudomonas putida* KT2440. We demonstrate that increasing CO_2_ levels boost root colonization by WCS417 in soil with relatively low C and nitrogen (N) content. Interestingly, these effects were associated with contrasting growth and resistance responses by the host plant, demonstrating that high atmospheric CO_2_ concentration can have profound and counterintuitive effects on plant growth and resistance due to altered rhizosphere interactions.

## Results

### Impacts of Atmospheric CO_2_ on Rhizosphere Colonization by Soil Bacteria Depends on Soil Quality and Bacterial Species

**FIGURE 1:**
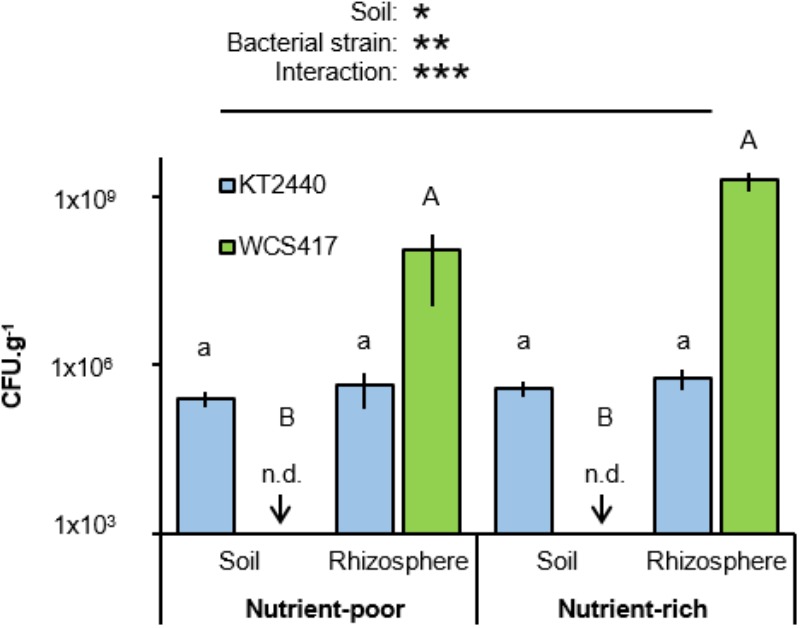
Effects of soil nutritional status on colonization by *P. simiae* WCS417 and *P. putida* KT2440. Bacteria were introduced into nutrient-poor soil or nutrient-rich soil at 5 × 10^7^ Colony forming units (CFU).g^−1^. CFU.g^−1^ of KT2400 (blue) and WCS417 (green) were determined after 4 weeks. Samples were taken from root-associated rhizosphere soil (Rhizosphere), or bulk soil without plants (Soil). Data represent mean CFU.g^−1^ values (± SE, *n* = 8). Asterisks on top of the graph indicate statistical significance of 2-way ANOVA (^∗^: 0.05 < *P* < 0.01, ^∗∗^: 0.01 < *P <* 0.001, and ^∗∗∗^: *P* < 0.001). Different letters of same font indicate statistically significant differences between soil-types for each strain (1-way ANOVA + Tukey multiple comparisons test; *P* < 0.05). n.d: not detected; bacterial titres were below the limit of detection.

C and N content are markers for soil quality (Gil-Sotres et al., 2005), which has a direct impact on the performance of PGPR (e.g., Egamberdiyeva, 2007; Agbodjato et al., 2015). To examine the importance of soil quality on rhizosphere colonization by two well-studied soil bacteria, Arabidopsis was cultivated either in artificial nutrient-poor soil (1:9 sand:compost; v/v) with low C- and N-contents, or in nutrient-rich soil (2:3 sand:compost; v/v) with relatively high C and N content (Table 1). Soils were inoculated with 5 × 10^7^ colony forming units (CFU).g^−1^ soil of *P. simiae* WCS417, a rhizosphere colonizer (Rainey, 1999; Zamioudis et al., 2014), or *P. putida* KT2440, a more generalist saprophytic soil colonizer (Weinel et al., 2002). Soil with and without Arabidopsis plants (accession Col-0) were left for 4 weeks before sampling for quantification of bacterial colonization through enumeration of CFU on selective agar medium. Two-way ANOVA of CFU values revealed a statistically significant interaction between soil and bacterial strain (*P* = 0.023; Figure 1 and Supplementary Table S1), indicating that the two strains colonize the soil-types and soil compartments to different extents. Indeed, statistical analysis by Tukey *post hoc* tests revealed that titres of the rhizobacterial strain WCS417 were significantly higher in the rhizosphere of Arabidopsis compared to those in plant-free bulk soil, where the level of colonization by this strain remained below the CFU detection limit (Figure 1). This rhizosphere-specific colonization by WCS417 was apparent in both soil-types (Figure 1). By contrast, the generalist saprophyte KT2440 colonized rhizosphere and bulk soil from both soil-types with equal efficiencies, although its levels of rhizosphere colonization remained orders of magnitude lower than that of WCS417 (Figure 1).

**Table 1:** C and N concentrations in nutrient-rich and poor-soil.

	Carbon (C)		Nitrogen (N)		C:N
Nutrient-poor	2.58%	−0.15	0.21%	−0.01	12.29
Nutrient-rich	18.78%	−0.48	0.37%	−0.03	51.02

**FIGURE 2:**
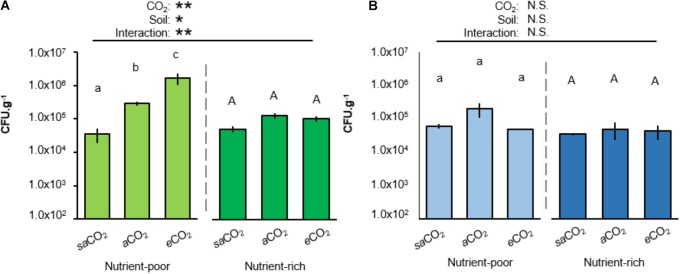
Impacts of atmospheric CO_2_ and soil-type on Arabidopsis rhizosphere colonization by *P. simiae* WCS417 **(A)** and *P. putida* KT2440 **(B)**. Bacteria were introduced at 5 × 10^7^ CFU.g^−1^ into nutrient-poor (left panels) or nutrient-rich (right panels) soil prior to planting Arabidopsis seeds. Rhizosphere colonization was determined after 4 weeks of growth at sub-ambient CO_2_ (200 ppm), ambient CO_2_ (400 ppm), or elevated CO_2_ (1200 ppm). Data shown represent mean CFU.g^−1^ (±SE, *n* = 10). Asterisks on top of the graph indicate statistical significance of 2-way ANOVA (^∗^: 0.05 < *P* < 0.01, ^∗∗^: 0.01 < *P <* 0.001, and ^∗∗∗^: *P* < 0.001). Different letters of the same font indicate statistically significant differences between CO_2_ conditions for each soil-type (Tukey multiple comparisons *post hoc* test, *P* < 0.05). Patterns of colonization with WCS417 were consistent over two independent experiments.

To examine whether atmospheric CO_2_ alters rhizosphere colonization by WCS417 and KT2440, Arabidopsis was cultivated for 4 weeks in both soil-types at *sa*CO_2_ (200 ppm), ambient CO_2_ (*a*CO_2_; 400 ppm) or *e*CO_2_ (1200 ppm) before quantification of rhizosphere colonization. Interestingly, in nutrient-poor soil, rhizosphere titres of WCS417 bacteria increased statistically from *sa*CO_2_ to *e*CO_2_, whereas this effect of CO_2_ was absent in nutrient-rich soil (Figure 2A). Furthermore, the statistically significant interaction between CO_2_ and soil-type indicates that the stimulating effect of CO_2_ on rhizosphere colonization by WCS417 depends on soil nutritional status (two-way ANOVA; *P* = 0.006; Figure 2A and Supplementary Table S2A). By contrast, rhizosphere titres of KT2440 were not statistically altered by CO_2_, soil-type, or the interaction thereof (two-way ANOVA; *P* = 0.541; Figure 2B and Supplementary Table S2B), indicating that the colonization by this saprophytic strain is unaffected by soil nutritional status and atmospheric CO_2_. Hence, the stimulatory impacts of atmospheric CO_2_ on rhizosphere colonization by soil bacteria depend on soil quality and bacterial species.

### Atmospheric CO_2_ Influences Plant Growth Responses to *P. simiae* WCS417 on Nutrient-Poor Soil

**FIGURE 3:**
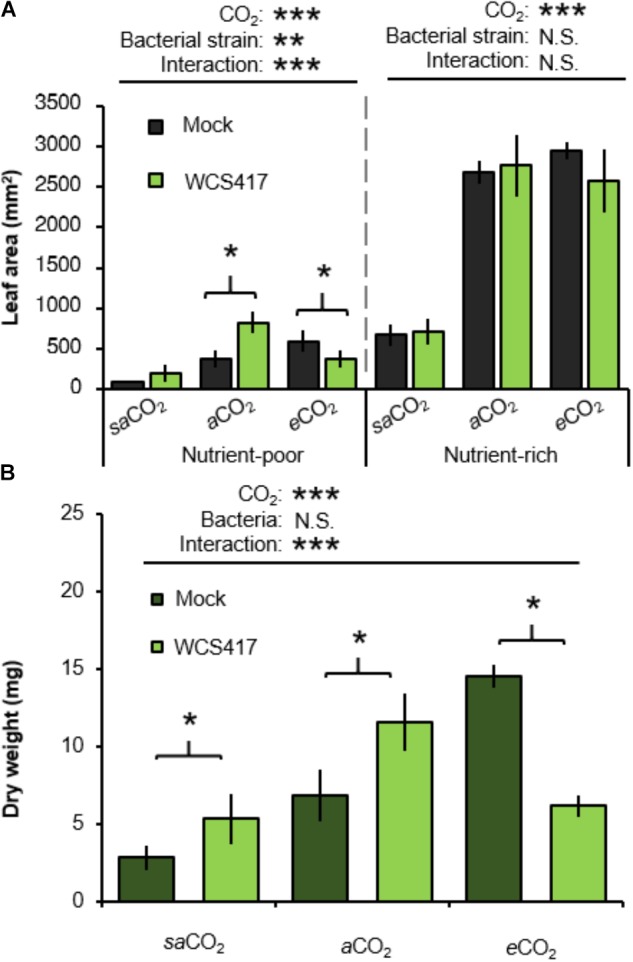
Effects of atmospheric CO_2_ and soil nutritional status on plant growth responses to *P. simiae* WCS417. **(A)** Effects of WCS417 on total leaf area of Arabidopsis at increased CO_2_ concentrations in nutrient-poor (left) and nutrient-rich (right) soils. Soil were inoculated with WCS417 (5 × 10^7^ CFU.g^−1^ soil), or or mock treated with MgSO_4_ prior to planting. Leaf area was quantified by image analysis after 4 weeks of growth. Shown are mean leaf areas (±SE, *n* = 10). **(B)** Effects of WCS417 root biomass at increased CO_2_ concentrations and in nutrient-poor soil. Data represent mean dry root weight values (± SE, *n* = 10). Asterisks on top of the graph indicate statistical significance of 2-way ANOVA (^∗^: 0.05 < *P* < 0.01, ^∗∗^: 0.01 < *P <* 0.001, and ^∗∗∗^: *P* < 0.001). Asterisks and parentheses indicate statistically significant differences between mock- and WCS417-treated soils (Student’s *t*-test; *P* < 0.05).

To assess the influence of CO_2_ on plant growth responses to rhizobacteria, control- (i.e., mock inoculated) and WCS417-inoculated plants were examined for rosette areas after 5 weeks of growth. In the absence of WCS417, rosette sizes increased statistically from *sa*CO_2_ to *e*CO_2_, which was apparent in both nutrient-poor and nutrient-rich soil (Figure 3A and Supplementary Table S3A). Furthermore, application of WCS417 did not influence the growth of plants on nutrient-rich soil (Figure 3A). This was confirmed by two-way ANOVA, which did not indicate a statistically significant interaction between bacterial treatment and CO_2_ (*P* = 0.432; Supplementary Table S3B). Conversely, in nutrient-poor soil, WCS417 had a statistically significant effect on rosette size and also showed a statistically significant interaction with CO_2_ by 2-way ANOVA (*P* < 0.001; Supplementary Table S3B). This indicates that the effects of WCS417 on shoot growth are dependent on atmospheric CO_2_ concentration. Subsequent *t*-tests revealed that WCS417 statistically increased rosette size at *a*CO_2_ and repressed at *e*CO_2_ (Figure 3A). Since PGPR have been reported to affect root and shoot growth differentially through impacts on auxin and cytokinin levels (Vacheron et al., 2013), we also determined root biomass. As is shown in Figure 3B, root dry weights in nutrient-poor soil mirrored the effects of WCS417 on rosette area on this soil-type: the bacteria increased root biomass at *a*CO_2_, while they increased root biomass at *e*CO_2_. As for the average rosette area, the effects of WCS417r on root biomass were statistically significant and showed a statistically significant interaction with CO_2_ (Supplementary Table S3C). Together, these results suggest that WCS417 has a plant growth-promoting effect at *sa*CO_2_ and *a*CO_2_, but that it reduces plant growth at *e*CO_2_.

### Atmospheric CO_2_ Influences Systemic Resistance Responses to *P. simiae* WCS417 on Both Nutrient-Poor and Nutrient-Rich Soil

**FIGURE 4:**
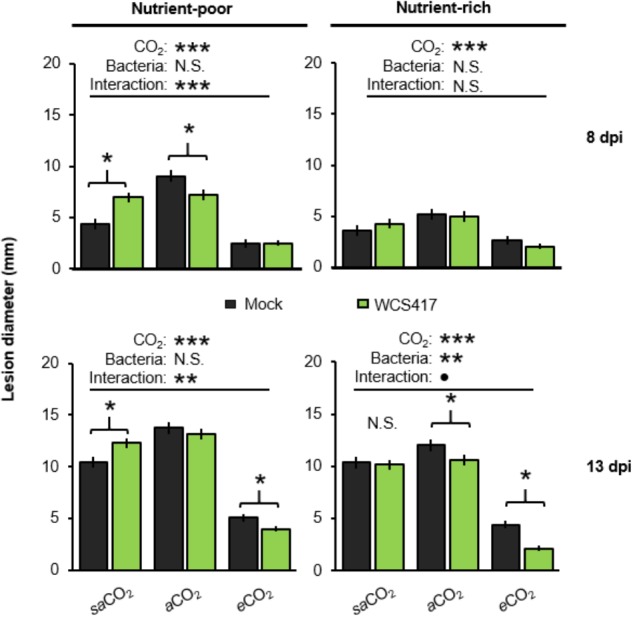
Effects of atmospheric CO_2_ and soil nutritional status on systemic resistance responses of Arabidopsis to *P. simiae* WCS417. Soil were inoculated with WCS417 (5 × 10^7^ CFU.g^−1^ soil), or mock treated with MgSO_4_ prior to planting. To quantify systemic resistance effects, 4-week-old plants were challenge-inoculated with *P. cucumerina* by applying 6-μL droplets of 5 × 10^6^ spores.mL^−1^ onto 4 fully expanded leaves per plant. Data shown are mean lesion diameters (± SE, *n* = 10) at 8 and 13 days post inoculation (dpi). Asterisks on top of the graph indicate statistical significance of 2-way ANOVA (: 0.1 < *P* < 0.05, ^∗^: 0.05 < *P* < 0.01, ^∗∗^: 0.01 < *P <* 0.001, and ^∗∗∗^: *P* < 0.001). Asterisks and parentheses indicate statistical differences (Student’s *t*-test; *P* < 0.05).

Arabidopsis develops induced systemic resistance (ISR) upon root colonization by WCS417 (Pieterse et al., 1996). Since WCS417 colonization of the Arabidopsis rhizosphere is CO_2_-dependent (Figure 2A), we examined impacts of CO_2_ on ISR. To this end, leaves of control- and WCS417-inoculated plants were challenge-inoculated with the necrotrophic leaf fungus *Plectosphaerella cucumerina*. Disease progression was quantified at 8 and 13 days post-inoculation (dpi) by lesion diameter in both nutrient-poor and nutrient-rich soil. For each time-point/soil-type combination (apart from 8dpi in nutrient-rich soil), two-way ANOVA revealed a statistically significant effect of CO_2_ on disease resistance (in each case *P* < 0.001; Supplementary Tables S4A–D), which manifested itself as increased resistance at *e*CO_2_ compared to *sa*CO_2_ and *a*CO_2_ (Figure 4). There was also a statistically significant interaction between bacterial treatment and CO_2_ in nutrient-poor soil which was apparent at 8 and 13 dpi in nutrient-poor soil, but was not significant at 13 dpi in nutrient-rich soil (two-way ANOVA; *P* < 0.001, *P* = 0.004, and *P* = 0.087, respectively; Supplementary Tables S4A–D). This indicates that the effects of WCS417 on systemic resistance depend on atmospheric CO_2_ concentration. Subsequent *t*-tests revealed that WCS417 reduced lesion diameters at both *a*CO_2_ and *e*CO_2_ in nutrient-poor and nutrient-rich soils, which was statistically significant at either 8 or 13 dpi (Figure 4). Surprisingly, at *sa*CO_2_, treatment of nutrient-poor soil with WCS417 statistically increased lesion diameters at both 8 and 13 dpi, suggesting induced systemic susceptibility (ISS). This response was absent when plants were grown on nutrient-rich soil at *sa*CO_2_, where WCS417 did not have a statistically significant effect on lesion diameter by *P. cucumerina* (Figure 4). Hence, the effect of WCS417 on systemic plant immunity varies from induced susceptibility to induced resistance, depending on the atmospheric CO_2_ concentration and soil nutritional status.

## Discussion

To date, only few studies have investigated effects of atmospheric CO_2_ on rhizosphere colonization by PGPRs. While previous work has shown that *e*CO_2_ increases bacterial and fungal biomass in the rhizosphere (Kassem et al., 2008), our study is the first to report effects of *sa*CO_2_ and *e*CO_2_ on rhizosphere colonization by selected soil bacteria. Procter et al. (2014) reported an increase in fungal species richness and enhanced relative abundance of selected fungi with *e*CO_2_, which varied according to soil-type (Procter et al., 2014). Furthermore, a grassland free air CO_2_ enrichment (FACE) experiment revealed that initial C accumulation occurred predominantly in arbuscular mycorrhizal fungi (AMF; Denef et al., 2007), which are symbiotic and rely on host-derived carbon (e.g., Lindahl et al., 2010). Although mycorrhizal root colonization is influenced by different factors than rhizobacterial root colonization, it is plausible that increased C deposition at *e*CO_2_ has more pronounced effects in C-poor soil-types, where root-associated microbes will be more reliant on plant-derived C. Indeed, the rhizobacterial WCS417 strain showed increasing rhizosphere colonization at rising CO_2_ concentrations, which was most pronounced in nutrient-poor soil (Figure 2). Moreover, the differential effects of CO_2_ on KT2440 and WCS417 help to explain why CO_2_ has been reported to have effects on some bacterial soil communities, while others remain unaffected (Rice et al., 1994; Ross et al., 1995; Kassem et al., 2008; Eisenhauer et al., 2012). Exactly what changes in rhizosphere chemistry drive these community effects, requires further research.

KT2440 was originally isolated from benzene-contaminated soils in Japan (Nakazawa and Yokota, 1973). Accordingly, it survives well in root-free bulk soils. However, this strain has also been reported to colonize the rhizosphere of plants, in particular of grasses (Molina et al., 2000). The rhizosphere of many grass species, such as maize, contain relatively high concentrations of aromatic benzoxazinoids (Neal et al., 2012). KT2440 is highly tolerant to the antimicrobial activity of benzoxazinoids and responds to these chemicals by positive chemotaxis (Neal et al., 2012), explaining why this strain is a strong colonizer of the maize rhizosphere. By contrast, KT2440 did not show increased colonization of the Arabidopsis rhizosphere in comparison to plant-free control soil (Figure 1), suggesting that KT2440 is not majorly influenced by the rhizosphere chemistry of Arabidopsis. WCS417, on the other hand, showed relatively high levels of colonization in the rhizosphere, but failed to sustain colonies in plant-free control soil (Figure 1), which is typical for a rhizobacterial species. WCS417 was originally isolated from the rhizosphere of wheat (Lamers et al., 1988) and has since been shown to colonize the rhizosphere of a wide range of plant species (Berendsen et al., 2015). Interestingly, a recent report has shown the iron-regulated secondary metabolite scopoletin in Arabidopsis root exudates selectively inhibits soil-borne pathogens, while ISR-inducing rhizobacteria, including WCS417, are highly tolerant to the antimicrobial effect of scopoletin (Stringlis et al., 2018). Hence, the recruitment and establishment of rhizosphere-colonizing bacteria not only depends on primary metabolites, but also on their sensitivity to secondary metabolites. The extent to which the exudation of scopoletin, and other possible rhizosphere chemicals, are influenced by atmospheric CO_2_ in Arabidopsis requires further investigation.

Rhizosphere colonization by PGPR promotes shoot and root development through different mechanisms (Lugtenberg and Kamilova, 2009). For instance, *Pseudomonas fluorescens* WCS365 has been shown to convert exuded tryptophan into the plant growth hormone auxin (Kamilova et al., 2006). In nutrient-poor soil, growth promotion by WCS417 was apparent under both *sa*CO_2_ and *a*CO_2_ (Figure 3). However, WCS417 repressed plant growth at *e*CO_2_ (Figure 3), indicating potentially pathogenic activity. This hypothesis is supported by the colonization data (Figure 2), which revealed >10 fold higher colonization of WCS417 at *e*CO_2_ compared to that at *a*CO_2_. It is tempting to speculate that such high densities at the root surface are perceived as hostile by the host immune system, triggering a growth-repressing immune response. The continuum between mutualism and pathogenic lifestyles is a recognized phenomenon for fungal endophytes (Schulz and Boyle, 2005) and other root colonizers (Bever et al., 2012). Interestingly, this plasticity is partially driven by environmental factors, including CO_2_ (Anderson et al., 2004; Schulz and Boyle, 2005). Although the relationship between plant-microbial mutualism and environmental factors remains poorly understood (Garrett et al., 2006, 2011; Johnson and Gehring, 2007), the growth repression by WCS417 at *e*CO_2_ was marked by relatively high levels of resistance against *P. cucumerina* (Figure 3). While this resistance appears to be an additive result of ISR and *e*CO_2_-induced resistance (Williams et al., 2018), it is plausible that these high levels of resistance are associated with costs to plant growth, which become apparent under nutrient-limiting conditions. ISR has been associated with priming of jasmonic acid and ethylene-controlled defenses (Pieterse et al., 2002). Even though priming is generally considered to be a low-cost defense strategy (van Hulten et al., 2006), the additive effect of *e*CO_2_ and ISR may result in constitutive up-regulation of inducible defenses that incur a detectable cost on plant growth under nutrient-limiting conditions. This hypothesis gains support from the observation that WCS417 only represses growth at *e*CO_2_ in nutrient-poor soil (Figure 3).

Our study has shown that two well-characterized soil bacteria display different rhizosphere behavior in response to changes in atmospheric CO_2_. Moreover, the plant responses to colonization by the rhizobacterial colonizing strain revealed a range of outcomes, including growth repression and induced systemic susceptibly. These findings demonstrate that predictions about impacts of global change and soil quality on crop performance need to take into account the complex interactions taking place in the rhizosphere. This outcome highlights the need for further research on the impacts of future global change on rhizosphere chemistry and the associated root microbiome.

## Materials and Methods

### Plant Cultivation and Growth Conditions

*Arabidopsis thaliana* (Arabidopsis), accession Columbia (Col-0) was cultivated in mx flow 6000 cabinets (Sanyo, United Kingdom) under ambient conditions (*a*CO_2_; 400 ppm, i.e., μL L^−1^), sub-ambient CO_2_ (*sa*CO_2_; 200 ppm), or elevated CO_2_ (*e*CO_2_; 1200 ppm). CO_2_ concentrations were chosen specifically to reflect two aspects of global change; 200 ppm was used as a post-glacial and pre-industrial atmospheric concentration, to imitate Arabidopsis’ ancestral habit (Beilstein et al., 2010; Beerling and Royer, 2011), and 1200 ppm was selected as worse case representative concentration scenario, as highlighted in the most recent intergovernmental panel on climate change report (IPCC, 2013). Growth chambers were supplemented with compressed CO_2_ (BOC, United Kingdom) or scrubbed with Sofnolime 797 (AP diving, United Kingdom) to maintain constant CO_2_ levels at indicated concentrations. Plants were cultivated under short-day conditions (8.5: 15.5 h light: dark; 20°C light, 18°C dark; 65% relative humidity). Seeds were stratified for 2 days (d) in the dark at 4°C and planted in 60-mL pots, containing a sand (silica CH52): dry compost (Levington M3) mixture, in a ratio of 2: 3 for nutrient-rich soil, or 1: 9 for nutrient-poor soil (v:v in both instances). Pots with plant-free control soil were set up and maintained under the same growth conditions. All pots were placed in trays to allow for bi-weekly watering. At 7 days after germination, seedlings were thinned to prevent crowding. To limit variation between different CO_2_ conditions, and compensate for pseudoreplication generated via chamber effects, experiments were conducted in identical climate chamber models, the exact same batches of seed and soil were used throughout each experiment. Furthermore, plant trays within each chamber were rotated weekly in a randomized fashion to counter positional effects.

### Soil Carbon (C) and Nitrogen (N) Concentrations

C and N concentrations in soil-types were determined by the complete combustion method followed by gas chromatography, using an ANCA GSL 20-20 Mass Spectrometer (Sercon PDZ Europa; Cheshire).

### Soil Treatment With Pseudomonas Simiae Wcs417 and Pseudomonas Putida KT2440 and Quantification of Bacterial Colonization

To determine impacts of CO_2_ on colonization of rhizosphere bacteria, yellow fluorescent protein (YFP)-expressing *P. simiae* WCS417 (Berendsen et al., 2012) was cultivated on selective Lysogeny broth (LB) agar (5 μg mL^−1^ tetracycline and 25 μg.mL^−1^ rifampicin). One YFP-fluorescent colony was selected for propagation in an overnight culture of liquid LB, containing the same selective concentrations of tetracycline and rifampicin. The medium was incubated in an orbital shaking incubator for 16 h at 28°C at 200 revolutions per minute (rpm). A similar method was employed for the cultivation of a green fluorescent protein (GFP)-expressing *P. putida* KT2440, which carries a stable chromosome-inserted PA_1/04/03_-RBSII-*gfp*mut3^∗^-T0-T1 transposon at a negligible metabolic cost (Dechesne and Bertolla, 2005). However, in this case, the bacteria were grown on minimal solid media (M9), after which one GFP-fluorescent colony was selected for propagation in LB liquid medium without selective antibiotics. Soils were inoculated with WCS417 or KT2240 bacteria by adding a bacterial suspension in 10 mM MgSO_4_ at a final density of 5 × 10^7^ CFU.g^−1^, or a mock treatment of 10 mM MgSO_4_ alone. Seeds were planted directly on the soil. Four weeks after germination, samples of root adhering rhizosphere soil and control soil (∼2 g) were collected, serially diluted and stamp-plated, using a 96-well Replica plater (Sigma-Aldrich, R2383) onto selective LB agar with tetracycline and rifampicin for WSC417, and M9 without antibiotics for GFP-expressing KT2240. Fluorescent colonies were enumerated using a Dark Reader DR195M Transilluminator (Clare Chemical) and normalized to sample weight. The colonization experiments (Figure 2) were repeated once with comparable results.

### Plant Growth Analysis

To determine the size of the plants, rosette area was estimated non-destructively from digital photographs (Canon EOS 500D) of rosettes, taken with a size standard. Image analysis involved converting pixels per rosette into area (mm^2^), using imaging software (Corel Paintshop Pro, ver. X7). To determine root growth, root material plus soil was collected and oven dried using an economy incubator 2 (Weiss Technik, United Kingdom; 60°C). Subsequently, roots were carefully extracted from the surrounding soil and weighed, using an analytical balance (Mettler Toledo AJ100).

### Induced Systemic Resistance Assays

To quantify WCS417-mediated ISR, plants were grown in soil with and without WCS417 bacteria as described above. After 5 weeks of growth, plants were challenge-inoculated with *P. cucumerina* (strain BMM). Lesion diameters were enumerated at 8 and 13 dpi and analyzed using Student’s test (*P* < 0.05). To ensure necrotrophic infection, *P. cucumerina* was applied by droplet inoculation (6 μL, 5 × 10^6^ spores mL^−1^) on 4 to 6 fully expanded leaves of plants (*n* = 8), as described previously (Pétriacq et al., 2016). Disease progression was determined by quantification of lesion diameters at 8 and 13 dpi, which correlates with fungal colonization disease progression (Pétriacq et al., 2016; Williams et al., 2018). Four lesion diameters per plant were averaged and treated as one biological replicate (*n* = 8). Differences in average lesion diameter between treatments were analyzed for statistical significance by ANOVA (using R, v. 3.1.2).

## Author Contributions

JT and DB conceived the project. AW, PP, TC, and JT planned the experiments. AW, TC, and PP performed the experiments. JT and DB provided reagents, equipment, and facilities. AW, PP, and JT analyzed the data. AW and JT wrote the paper with feedback from all co-authors.

## Conflict of Interest Statement

The authors declare that the research was conducted in the absence of any commercial or financial relationships that could be construed as a potential conflict of interest.
