# Supplementary material for: Impacts of Atmospheric CO2 and Soil Nutritional Value on Plant Responses to Rhizosphere Colonization by Soil Bacteria
Source: Front Plant Sci. 2018 Oct 22;9:1493. doi: 10.3389/fpls.2018.01493 (PMC6204664; doi:10.3389/fpls.2018.01493)
Supplement: Supplementary file 1 [file Data_Sheet_1.pdf]

Supplemental Tables

Table S1: Two-way ANOVA of bacterial titres by soil and bacterial strain (KT2440 or WCS417)

Test	F-value	P-value	Df
Bacterial strain	5.389	0.00344	3
Soil	4.580	0.03882	1
Interaction (Soil x Bacterial strain)	3.561	0.02296	3

Df of the residuals = 38

Table S2A: Two-way ANOVA of bacterial inoculation (*Pseudomonas fluorescens*) by atmospheric CO_2 and soil-type.

Test	F-value	P-value	Df
CO_2	6.925	0.00210	2
Soil	7.017	0.01057	1
Interaction (CO_2 x Soil)	5.543	0.00647	2

Df of the residuals = 54

Table S2B: Two-way ANOVA of bacterial inoculation (*Pseudomonas putida*) by atmospheric CO_2 and soil-type.

Test	F-value	P-value	Df
CO_2	0.542	0.255	2
Soil	1.587	0.160	1
Interaction (CO_2 x Soil)	0.379	0.341	2

Df of the residuals = 50

Table S3A: Two-way ANOVA of rosette size by atmospheric CO_2 and bacterial strain (WCS 417) in nutrient-poor soil.

Test	F-value	P-value	Df
CO_2	39.243	1.37x10e-10	2
Bacterial strain	8.527	0.00545	1
Interaction (CO_2 x Bacterial strain)	34.497	8.27x10e-10	2

Df of the residuals = 55

Table S3B: Two-way ANOVA of rosette size by atmospheric CO_2 and bacterial strain (WCS 417) in nutrient-rich soil.

Test	F-value	P-value	Df
CO_2	61.516	5.7x10e-14	2
Bacterial strain	0.380	0.540	1
Interaction (CO_2 x Bacterial strain)	0.853	0.432	2

Df of the residuals = 48

Table S3C: Two-way ANOVA of root weight by atmospheric CO_2 and bacteria (WCS 417) in nutrient poor soil.

Test	F-value	P-value	Df
CO_2	14.246	0.000123	2
Bacteria	0.688	0.416060	1
Interaction (CO_2 x Bacterial strain)	15.065	8.75x10e-5	2

Df of the residuals = 21

Table S4A: Two-way ANOVA in systemic resistance against *Plecosphaerella cucumerina* by atmospheric CO_2 and bacterial treatment, at 8 dpi in nutrient-poor soil.

Test	F-value	P-value	Df
CO_2	95.120	<2x10e-16	2
Bacterial treatment	2.424	0.126	1
Interaction (CO_2 x Bacterial treatment)	16.772	3.38x10e-6	2

Df of the residuals = 46

Table S4B: Two-way ANOVA in systemic resistance against *Plecosphaerella cucumerina* by atmospheric CO_2 and bacterial treatment, at 8 dpi in nutrient-rich soil.

Test	F-value	P-value	Df
CO_2	33.568	3.36x10e-10	2
Bacterial treatment	0.031	0.860	1
Interaction (CO_2 x Bacterial treatment)	1.757	0.182	2

Df of the residuals = 54

Table S4C: Two-way ANOVA in systemic resistance against *Plecosphaerella cucumerina* by atmospheric CO_2 and bacterial treatment, at 13 dpi in nutrient-poor soil.

Test	F-value	P-value	
CO_2	194.132	<2x10e-16	2
Bacterial treatment	0.055	0.81613	1
Interaction (CO_2 x Bacterial treatment)	6.301	0.00372	2

Df of the residuals = 48

Table S4D: Two-way ANOVA in systemic resistance against *Plecosphaerella cucumerina* by atmospheric CO_2 and bacterial treatment, at 13 dpi in nutrient-rich soil.

Test	F-value	P-value	
CO_2	176.435	<2x10e-16	2
Bacterial treatment	11.598	0.00127	1
Interaction (CO_2 x Bacterial treatment)	2.559	0.08691	2

Df of the residuals = 53
